# Got milk? Not in public!

**DOI:** 10.1186/1746-4358-3-11

**Published:** 2008-08-04

**Authors:** Jacqueline H Wolf

**Affiliations:** 1 Department of Social Medicine, College of Osteopathic Medicine, Ohio University, Athens, Ohio, USA

## Abstract

The American focus on the sexual purpose of breasts, rather than the physiological function of breasts, has serious public health consequences. Discomfort with breastfeeding in public lowers breastfeeding rates, which in turn negatively affects women's and children's short- and long-term health.

## Debate

When I was growing up in the 1960s my best friend's father was an obstetrician. This was the heyday of formula feeding and my friend's father, even with his vast experience in women's reproductive health, had virtually no experience with breastfeeding. His expertise continued to be so negligible, in fact, that when my friend nursed her newborn son in front of him in the early 1980s – this is his first grandchild being nursed – he watched for a minute and then he said to his daughter, "That will ruin your breasts, you know." When my friend called to report this comment to me sometime later, she was still bewildered. She asked me: "What does he think breasts are *for*?"

Her story captures the essence of why breastfeeding in public is such a controversial topic in American culture. Americans think breasts are primarily for enhancing sexual activity, which results in widespread discomfort when they are reminded that breasts go into babies' mouths. While *our *culture defines breasts primarily as enhancing sexual appeal, other cultures emphasize the "sexiness," if you will, of *other *body parts: buttocks, hips, shoulders, feet. It varies according to culture, since sexual attractiveness is always culturally defined.

Yet the American obsession with breasts is so unique that it is often puzzling to people from other cultures. One of my graduate students who conducted field work in Samoa reported to me that when she told a Samoan woman that American men like to suck on women's breasts, the Samoan was amazed. She asked my student, apparently with genuine interest and concern, "Do American men like to pretend they're babies?" That is how strictly Samoans define the purpose of women's breasts. In many countries, breastfeeding in public is as mundane an activity as public conversation; no one is concerned when women use their breasts in public for their primary biological function: to feed babies.

My purpose today is not to argue that we should think more like Samoans, but to point out that the American obsession with breasts has consequences most Americans fail to consider: ready access to human milk is vital to babies' short- and long-term health. We are all affected by our culture's sexual emphasis on breasts and our consequent discomfort with breastfeeding in public. While people from other cultures often find this controversy inexplicable, the reasons for the controversy are obvious to Americans – even those of us who fully support breastfeeding in public. We understand that many equate public breastfeeding with lewd behavior. However, equating breastfeeding with vulgarity has dire consequences; this attitude lowers the country's breastfeeding rates which in turn affects women's and children's short and long-term health. If women are made to feel uncomfortable with public breastfeeding, breastfeeding becomes difficult, if not impossible, to sustain. Women who have successfully breastfed for long periods of time know that unless women can feed their babies anytime, anywhere, they're going to end up housebound. And it's the rare American woman who is willing to be housebound for months on end. So, many women give up breastfeeding early on and opt for the bottle.

Here are the statistics: 70% of new mothers initiate breastfeeding. But all initiation means is breastfeeding once before hospital discharge. Of far greater meaning is the breastfeeding exclusivity rate, which is terrible. By the time American babies are six weeks old, 53% of breastfeeding mothers have introduced at least some infant formula to their babies' diet and by six months, 90% [1]. Fewer than 18% of mothers who initiate breastfeeding are giving their babies *any breast milk at all *at the end of the minimal year recommended by the American Academy of Pediatrics.

One reason that rates are so high *initially *and then plummet almost immediately when it comes to exclusivity and duration is because most women are *not c*omfortable breastfeeding in public because the public is not comfortable seeing them breastfeed. That is a fundamental problem because human milk is low in fat and it is especially low in protein, much lower, for example, than cows' milk. That means human babies are almost constant feeders by design – which is why women in so many cultures literally wear their babies on their bodies, for easy access so they can feed often. In the U.S., people who oppose breastfeeding in public often argue, "What's the problem? Feed the baby before you leave the house. There's no reason, with a little planning, to breastfeed in a restaurant or at the mall." This insistence that babies should only be breastfed behind closed doors demonstrates a fundamental lack of understanding of both the composition of human milk and babies' needs. Babies have to nurse while they are out and about due to the nature of human milk.

This insistence that babies should only be breastfed behind closed doors exposes our split personality on breastfeeding. We insist breastfeeding is a good thing to do. And, we insist it is an offensive thing to do.

Our squeamishness with breastfeeding in public has consequences we refuse to acknowledge. Hurricane Katrina is a prime example of an unacknowledged consequence of our culture's revulsion at breastfeeding in public. We all watched with horror as New Orleans drowned two years ago. For days, Katrina and its aftermath were the only items in the news. And, whenever there is a news story of that magnitude there are always a lot of sidebars to the story. Remember? Katrina exposed America's class and racial divisions. We heard stories about inefficient government agencies, abandoned pets, lethal mold.

But do you know what story I kept looking for and never found? What happens to formula fed babies during a disaster when mothers cannot buy infant formula and they do not even have access to water? And there was ample opportunity to have a sidebar that pondered those awful questions. Some of the most memorable film clips coming out of New Orleans in 2005 pictured frantic mothers clutching their barely conscious, dehydrated babies.

Discussing our culture's attitude toward breastfeeding in relation to the Katrina disaster would have been a tremendous public service. Think about how much our discomfort with breastfeeding would have been mitigated if one of the Katrina reports contrasted the convenience and dependability of breastfeeding with the difficulty and unreliability of formula feeding. Imagine: images of mothers' breasts saving babies' lives. That would have been a national revelation. We talk about the importance of breastfeeding, yet we're a formula feeding culture. It seemed perfectly natural to all the reporters and much of the viewing audience during Katrina that mothers were hysterical and babies were dehydrated because there was not enough infant formula available. The entire nation seemed to be saying, "Of course that's a consequence of a hurricane." No reporter thought to ask, "Why aren't these women breastfeeding?" No reporter thought to ask, "What roadblocks have we constructed as a nation that would dissuade women from breastfeeding and put their babies through this completely avoidable horror?"

To those of us who work on breastfeeding, the "issue" of breastfeeding in public is a periodic amusing and frustrating annoyance. However, we have to start treating it as more than that. The negative attitude toward public breastfeeding is a cornerstone of low breastfeeding rates and a basis of our persistently formula feeding culture. Aside from all the mothers who quickly learn to use infant formula because they are embarrassed by their hungry babies when there is no private space to breastfeed, women in the U.S. often fail at breastfeeding because they do not have adequate opportunity to observe other women breastfeeding. Breastfeeding is not intuitive, it is a learned behavior. In other words: Breastfeeding in our culture is deemed a private bodily function when – for many reasons, all having to do with infant and maternal health – it *should *be a public one.

## Competing interests

The author declares that they have no competing interests.
